# Medically Unexplained Symptoms (MUS): Faults and Implications

**DOI:** 10.3390/ijerph16071247

**Published:** 2019-04-08

**Authors:** Michiel Tack

**Affiliations:** Independent researcher, Sint-Laurentiusstraat 87, 9700 Oudenaarde, Belgium; tackmichiel@gmail.com

**Keywords:** medically unexplained symptoms (MUS), functional somatic syndromes (FSS), chronic fatigue syndrome (CFS), fibromyalgia, somatic symptom disorder (SSD)

## Abstract

The classification of medically unexplained symptoms (MUS) could have negative consequences for patients with functional somatic syndromes (FSS). By grouping related but distinct syndromes into one label, the MUS classification fails to inform clinicians about their patients’ health condition. In research settings, the MUS classification makes patient samples more heterogeneous, obstructing research into the underlying pathology of FSS. Long-term studies have shown that MUS are often appraised as medically explained symptoms at follow-up and vice versa, raising doubts about the reliability of this distinction.

In their recent paper, Guo et al. evaluate commonalities across medically unexplained symptoms (MUS) [1]. The justification of this classification has not been adequately discussed in the scientific literature.

The main argument for MUS is the significant symptom overlap across several medically unexplained syndromes. According to Guo et al. [1], a patient with unexplained fatigue and widespread pain might be diagnosed with chronic fatigue syndrome (CFS), fibromyalgia (FM) or somatic symptom disorder (SSD) depending on the clinical setting. While correct, this diagnostic overlap is mostly a consequence of how these syndromes have been defined. FM, for example. “remains a valid construct irrespective of other diagnoses” [2]. This means a CFS patient can be diagnosed with comorbid FM without diagnostic confusion. In a community-based sample, 15% of CFS patients also met diagnostic criteria for FM [3]. Similar rates have been found in rheumatic conditions such as systemic lupus erythematosus (SLE) or rheumatoid arthritis (RA), indicating that this diagnostic overlap is not restricted to functional somatic syndromes [4]. Studies have shown that CFS patients with comorbid FM have more impairments [5,6] and a worse prognosis [7] than CFS patients without FM. The influence of comorbid FM on the prognosis of CFS persists after controlling for multiple nonspecific symptoms. This is inconsistent with the hypothesis that CFS and FM represent different manifestations of a single underlying syndrome. While CFS and FM are related conditions, research has indicated biological differences between the two [8,9].

A patient with CFS or FM might also be diagnosed with SSD. This is a consequence of the broad SSD criteria defined in the fifth edition of the Diagnostic and Statistical Manual of Mental Disorders (DSM-V), which, according to some commentators, “open the floodgates to the over-diagnosis of mental disorder” [10]. The diagnosis of SSD merely requires that a persistent physical symptom is accompanied by disproportionate health-related thoughts and behaviors [11]. While the definition of somatization disorder in the DSM-IV required a history of multiple medically unexplained symptoms before the age of 30, the diagnosis of SSD can essentially be made when a patient worries excessively about his or her medical condition. Consequently, patients with CFS or FM might be diagnosed with SSD, yet the same is true for “explained” medical conditions such as RA, SLE or liver cirrhosis [12]. Instead of questioning the legitimacy of CFS or FM, a large diagnostic overlap with SSD challenges the DSM-V case definition as it risks mislabelling patients as mentally ill. Empirical research has questioned the validity of SSD in patients with functional somatic syndromes such as FM [13].

If the main difficulty faced by clinicians is diagnostic confusion and a need for better differentiation between functional somatic syndromes, then stricter diagnostic criteria seem warranted. Disregarding all differences between these health conditions by creating one large MUS classification will reinforce rather than solve this problem. CFS offers an example that a more accurate case definition does not necessarily require a better understanding of the underlying pathology. Instead of fatigue, the hallmark symptom of CFS is now considered to be post-exertional malaise (PEM), a significant symptom exacerbation each time patients exceed their current energy limit [14]. PEM helps clinicians to differentiate CFS patients from patients with idiopathic chronic fatigue [15] or multiple sclerosis [16] and predicts a worse prognosis [17].

While the benefits of the MUS classification are unclear, the risks and faults are evident. Due to PEM, CFS patients might respond differently to an exercise regime than patients with pain syndromes [18]. By grouping both types of patients into one clinical entity, MUS fails to provide therapists with the necessary information to offer the most adequate care.

Similar problems might arise in research settings. By making patient samples more heterogeneous, MUS risks obstructing research into the underlying pathology of functional somatic syndromes. Throughout medical history, the trend has been towards subgrouping and better differentiation of seemingly similar health conditions. Cancer, for example, was once considered to be a single disease but is now divided into multiple clinical entities [19]. A similar evolution can be found in current research on Alzheimer’s disease [20] or SLE [21]. The MUS classification seems to be an anachronistic exception to this rule.

If up to 50% of patients in hospital-based care have MUS, as Guo et al. [1] claim, one might question whether anything useful can be said about the etiology of such a broad and heterogeneous group of patients. No reason has been provided for why the numerous health complaints modern medicine fails to explain would have anything in common. The common factor between MUS patients seems to be the limitations of our medical technology and understanding, rather than the underlying condition of these patients. Additionally, MUS are often appraised as medically explained symptoms at follow-up and vice versa, raising doubts about the reliability of this distinction [22,23]. 

Finally, Guo et al.’s claim that correcting misinterpretations of somatic sensations could be seen as a preventive strategy of MUS is a contradiction in terms. If the authors truly believe that misinterpretations of ordinary somatic sensations are responsible for MUS, then they should not label these symptoms as unexplained but instead as psychiatric.
